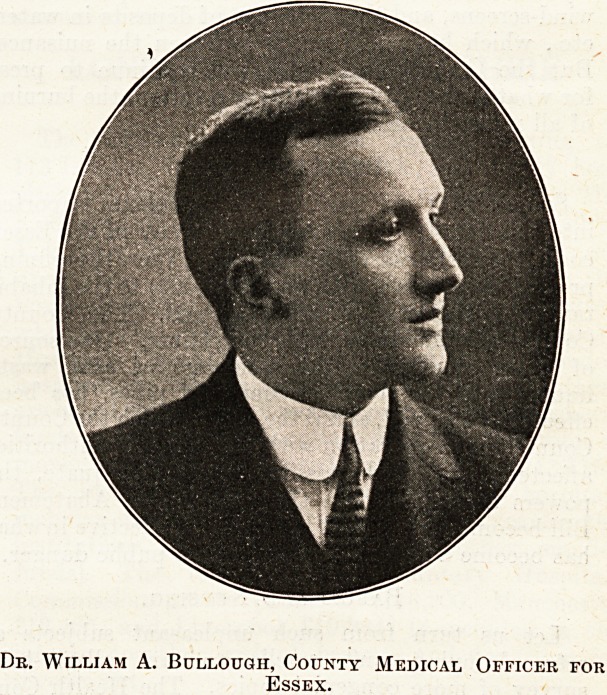# The Public Health: Interviews with Local Authorities—Essex

**Published:** 1923-12

**Authors:** 


### THE PUBLIC HEALTH.

### INTERVIEWS WITH LOCAL AUTHORITIES.

### XIV.—THE COUNTY OF ESSEX.

If you have thought of Essex as an agricultural county, with a quiet county-town of 20,000 inhabitants as its centre and seat of county government, you must think again. Better still, you should seek out, as we have done, the Chairman of the County Health Committee, Alderman S. W. Robinson, and the County Medical Officer, Dr. W. A. Bullough, and let them unfold to you some of the varied characteristics of the county and some of the health problems which are thereby involved. It is like looking through a kaleidoscope. Here one set of problems, there another; here and there some fresh groupings and arrangements. To the mind's eye of a Londoner, at any rate, a host of different pictures is conjured up by such names as Barking and Tilbury; Epping; Wanstead; Clacton and Frinton; Leyton and Walthamstow; Dunmow and Braintree and Harwich. No inconsiderable task, therefore, confronts Alderman Robinson and his Committee, and absorbs the energies of a very energetic Medical Officer and his staff; it is that of looking after the health of a county which comprises urban areas, residential suburbs, wide agricultural districts, small manufacturing centres, seaside resorts, and a seafaring, fishing and dockside population. Something of everything, you see. And the county has a population which, after eliminating the inhabitants of the county boroughs, numbers 920,000.

[*Cornhill*

Alderman S. W. Robinson, Chairman of the Essex Health Committee.

Dr. William A. Bullough, County Medical Officer for Essex.

#### London Problems

One-half of this population lives in the south-western corner of the county immediately adjacent to the metropolis. Leyton and Walthamstow together have a population of over a quarter of a million people. It is not surprising—as indeed the fact of our interview taking place in the Essex County's London offices in Finsbury Circus reminded us—that the health problems for the county authority have their greatest density in the outskirts of London. There is first the Housing problem. The county health authorities have interested themselves in a scheme of town-planning to put a stop to the haphazard building of which there is so great a danger in the district north of the Thames, where masses of population are tending to congregate. Unless such a scheme is pressed forward houses will be erected on low-lying, unsuitable sites, with consequent ill-effects to the workers and their families. It is soundly conceived, and it is sad to record that for the time being it is frustrated by the cold hand of finance. Meanwhile, it is pleasant to note the Medical Officer's view that so much of what has been written about jerry-built Council houses elsewhere cannot refer to this county, where the Council houses are well built, well drained and ventilated. As a significant contribution towards the solution of the housing difficulty, the 1921 statistics show that 10,218 houses were rendered fit in consequence of informal action by the local authority, and that notices were served requiring defects to be remedied on owners of 5,300 houses, such defects being remedied in the great majority of cases.

#### The Dumping of Refuse

This is an ever-present problem arising out of the fact that a thickly-populated part of the county is so close to London. So long ago as 1911 it was estimated that London's refuse—consisting of house waste, road scrapings, gully sludge, fish and other market garbage, and other equally unpleasant matter—amounted to about 1,250,000 tons a year, of which only one-third was being burnt in destructors, leaving about 800,000 tons to be removed to tips. The greater part of this is dumped on Essex marshes, and there ensue smells, smoke, flies, rats, dirty paper scattered by the wind, verminous trouble and infection of children playing on or near the dumps and pollution of streams. This has been, and still is, a cause of grave anxiety to Essex health authorities. Conferences with the London sanitary authorities and with the Ministry of Health have resulted in measures requiring the dumping of the refuse in layers covered with earth or lime, the provision of wind-screens, and theavoidance of deposits in water, etc., which have done much to lessen the nuisance. But the County Committee will continue to press for what is the only satisfactory solution, the burning of all refuse in destructors.

### Deadly Fumes.

Some eighty per cent. of the petroleum imported into this country is said to enter by way of the Essex coast, the north side of the Thames. There the refining process takes place, with serious results to the inhabitants of districts for miles around. The County Council are inundated with complaints. One source of great annoyance, the discharge of acid waste liquors into the river by concealed pipes, has been effectively stopped, and if the action which the County Council, in co-operation with the sanitary authorities affected, now find possible is not adequate, the powers available when the new Smoke Abatement Bill becomes law will, it is hoped, be effective in what has become a serious nuisance and a public danger.

### Babies and Nursing.

Let us turn from such unpleasant subjects as refuse-dumping and smells to an all-too-brief survey of more congenial topics. The Health Committee do well to pride themselves on the fact that their infant mortality figure was down to 55.5 in 1922, a figure showing a wonderfully favourable comparison with that of 77 for England and Wales. Two districts, Burnham-on-Crouch and Frinton-on-Sea, are able to make the healthy boast that in 1922 no deaths of infants under one year of age occurred. They seem to know in Essex "How to take care of Baby," to quote the title of a much-appreciated pamphlet used in connection with the excellent maternity and child welfare work. Grants to the voluntary child welfare committees, and to the County Nursing Association, lectures to midwives, baby shows, the supply of milk to expectant and nursing mothers, all play their beneficent part in this scheme of saving child life. The Chairman of the Health Committee impressed on us the importance of the work of the district nurse-midwives in promoting the health and well-being of the community. As midwives they were the subject of not a single complaint during the year; as district nurses they co-operate with the County Council's whole-time health visitors in following up defects discovered among school-children, in the care and after-care of tuberculous patients, and so on. Truly excellent work. There are five members of the County Council on the executive of the County Nursing Association.

### Treatment of Tuberculosis.

The Essex County Council have given this matter very close attention during the past four years, particularly in respect to the provision of institutional treatment. This is very evident from the fact that in 1919 they provided 100 beds for tuberculous patients, whereas at the date of our interview 320 beds were occupied.

### Co-operation.

They believe in co-operation in Essex, and the relations of the County Medical Officer with the local medical officers and general practitioners and others are excellent. In the all-important matter of milk, for instance, there is co-operation with the various sanitary authorities in the attempts to securec lean dairies and cowsheds (we note, in passing, that Mr. A. Marsh, the County Sanitary Inspector, is also Dr. Bullough's chief clerk); co-operation with the Essex County Farmers' Union, to whose excellent propaganda in the matter of milk production the Chairman and Dr. Bullough paid tribute; and with the East Anglian Institute of Agriculture, which undertakes the bacteriological examination of milk. For further examples of this spirit of co-operation, our attention was drawn to the work of the county laboratory, to which the general practitioners freely resort; the London and Home Counties' joint scheme for fighting venereal disease; the arrangements with the London County Council whereby in an emergency one may ring up their smallpox expert direct (Hop 5000); and the visits of Sir Henry Gauvain to the children undergoing treatment for surgical tuberculosis. The Health Services undertafien by the County Public Health and County Education Committees are already, as from the date of Dr. Bullough's appointment in April, 1919, consolidated in one department under his supervision as County Medical Officer and School Medical Officer, with obvious advantages.

### Honorary Consultant in Health Matters.

The Essex County Council showed their appreciation of Dr. John C. Thresh's services as County Medical Officer for Essex for about thirty years by appointing him as Honorary Consultant in County Public Health matters. Dr. Thresh's work in connection with water supplies, sewage disposal, prevention of rivers, pollution, etc., is so well known as to need no further comment. Dr. Thresh maintains his great interest in Essex and recently he has given valuable assistance in connection with the augmentation of the water supplies for Chelmsford Borough and the Southend County Borough.

### Co-ordination: Theory and Practice.

We have left too little space for the big outstanding feature of health administration in the County of Essex. Many county authorities may, and do, express themselves with some hesitation on the subject of bringing all health services, including those of the Poor Law, under one co-ordinated control. But Alderman Robinson unhesitatingly expressed his own personal view that that is the road along which we must travel. And, indeed, the scheme of combined medical and sanitary services which has been built up in the county during Dr. Bullough's four short years of office is an illustration, wholly admirable, of what has already been accomplished in the county in the direction of co-ordination, without any drastic changes or alterations of statutory functions.

### The Combined Medical Service.

The object of the scheme is to secure by an agreement between the local sanitary authorities and the County Council that one Medical Officer, who must possess the Diploma of Public Health, shall, in each sanitary district, act in the dual capacity of local Medical Officer of Health, directly responsible to the local sanitary authority for all duties usually involved in such an appointment, and as Assistant County Medical Officer working under the supervision of, and responsible to, the County Medical Officer for the duties of Tuberculosis Officer, School Medical Inspector, Child Welfare, etc. The arrangements are in force and working with the utmost smoothness in twenty-four sanitary districts (combined into nine groups), containing over a quarter of a million of the population. One of the officers so appointed says: "I have not found any instance where my duties as Assistant County Medical Officer have been incompatible with those of local M.O.H." Says another: "I can think of no disadvantages"; and yet another: "It is the best thing that has happened in the county since I have known it."

### Absence of Friction.

It is claimed in support of the policy that there is avoidance of overlapping and unnecessary travelling, and a consequent saving of expense; that the prestige of the Medical Officer is raised; that he is better known to parents and doctors; that the home is not unnecessarily invaded; and that the continuity of supervision and treatment of child life, and a common public health policy, are maintained throughout the area. That the personal element must count for much in the smooth working of a scheme in which one man serves two masters cannot be gainsaid. It speaks much for the wisdom and sanity of outlook of Alderman Robinson and Dr. Bullough, and the several medical officers, that there has been a complete absence of friction.

## Figures and Tables

**Figure f1:**
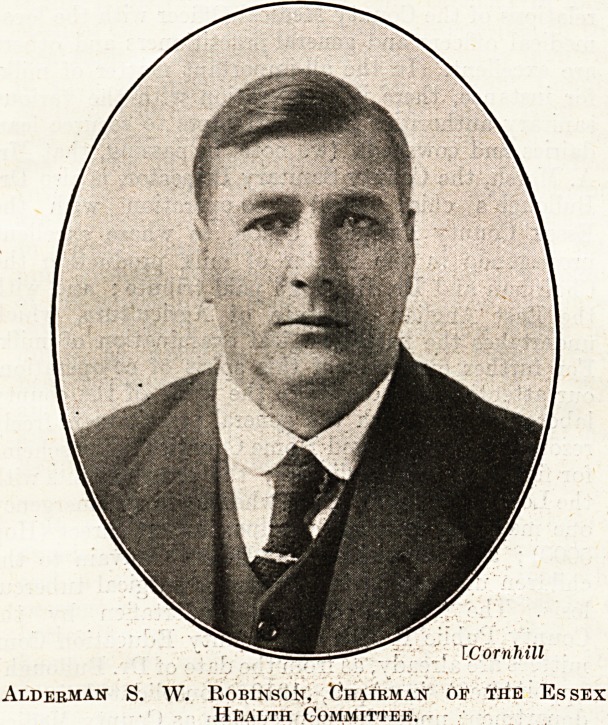


**Figure f2:**